# Species adulteration in raw milk samples using polymerase chain reaction-restriction fragment length polymorphism

**DOI:** 10.14202/vetworld.2018.830-833

**Published:** 2018-06-22

**Authors:** Rania M. Ewida, Doaa Safwat M. Abd El-Magiud

**Affiliations:** 1 Department of Food Hygiene (Milk Hygiene), Faculty of Veterinary Medicine, New Valley Branch, Assiut University, Assiut 71526, Egypt; 2 Department of Forensic Medicine and Toxicology, Faculty of Veterinary Medicine, New Valley Branch, Assiut University, Assiut 71526, Egypt

**Keywords:** buffalo’s milk, *cyt b* gene, Hinf I enzyme, medicolegal, milk adulteration, polymerase chain reaction-restriction fragment length polymorphism

## Abstract

**Background and Aim:**

Milk adulteration is pivotal because it leads to worse effects in public health as human adverse reactions with clinical signs ranged from gastrointestinal signs to anaphylactic shock. This study was carried out to estimate the prevalence of adulteration in buffalo’s milk sold in Assiut City, Egypt.

**Materials and Methods:**

A total of 50 raw buffalo’s milk samples were collected and examined for adulteration by addition of cow’s milk. The examination carried out by applying polymerase chain reaction-restriction fragment length polymorphism technique using *cytochrome b (cyt b)* gene primers and Hinf I enzymes. The size of target gene was 360 bp in both animal species and amplicon can be digested using Hinf I enzyme, this restriction enzyme divided the essential band to clear three bands at 360, 210, and 150 bp in cows’ milk, while, the enzyme could not be cleaved the amplicon in buffalo’s samples.

**Results:**

The obtained results cleared that the incidence of adulteration of buffalo’s milk very high percentage reaches 90%.

**Conclusion:**

It could be concluded that the raw buffalo’s milk sold in Assiut City subject to fraudulent practice and thus can lead to public health hazards.

## Introduction

Milk is considered as the most nearly perfect food; it has big value to children as well as the adult because it has many components as protein, minerals, and vitamins. Adulteration of milk is occur by adding inferior substance or removal of one or more essential components of it [1], and it is of a great for economics and public health hazard.

Milk adulteration has many forms; the most common form is the mixing of different types of milk species as addition of cow’s milk to buffalo’s one. Using cow’s milk in adulteration may be due to it is cheaper and greater production of milk from cows in comparison with buffalos in some farm [2]. This type of adulteration must be stopped because the cow’s milk protein leads to allergy to some people especially in children; in addition, it is responsible for human adverse reaction. The clinical signs of cow milk allergy were cutaneous, gastrointestinal, respiratory, and anaphylactic shock [3,4]. Moreover, cow’s milk addition must be avoided due to religious and ethical objections [5] or due to governmental regulation [6]. Therefore, the species identification becomes an important issue in current food safety requirement.

Many different techniques used for species identification as chemical [7], immunological [8], electrophoretic [9], chromatographic [10], reversed-phase high-performance liquid chromatography, and ELISA [11,12]. Recently, molecular techniques have used for species identification, and it has been proved due to the simplicity, sensitivity, repeatability, and reproducibility [13,14].

Polymerase chain reaction-restriction fragment length polymorphism (PCR-RFLP) assay is one of the recent molecular techniques which applied in species identification in milk and milk products. Besides, the previous advantage of molecular techniques, PCR-RFLP has lower cost in comparison with other methods as real-time PCR. The RFLP profile can be obtained in few hours [15,16].

The present study aimed to estimate the prevalence of adulterated buffalo’s milk samples by cow’s milk sold in Assiut markets, Egypt, using PCR-RFLP technique.

## Materials and Methods

### Ethical approval

Ethical approval is not required to pursue this type of study.

### Samples and study area

A total of 50 raw buffalo milk samples were collected from different dairy shops and street vendors located in Assiut City, Egypt. According to the information provided by the vendors, all samples contained pure buffalo’s milk. All samples were transported immediately to the laboratory in the icebox. From each milk sample, we collected (50 ml) in a sterile screw-capped bottle. The samples were stored at −20°C until DNA extraction.

### PCR

This part has been done in Molecular Biology Research Unit (Certified ISO/IEC: 17025-2005).

### DNA extraction

DNA extraction was carried out using Patho Gene-spin^™^ DNA/RNA Extraction kit (ISO 9001/14001) for the samples, positive control (obtained from dairy farm milk), and negative control (bacterial strain of *E. coli*).

### DNA amplification

DNA amplification was carried out using specific primers to detect the *cytochrome b (cyt b*) gene of mitochondrial DNA according to Parson *et al*. [17]. L14816 (5\ CCA TCC ACC ATC TCA GCA TGA TGA AA) and H15173 (5\ CCC CTC AGC ATG ATA TTT GTC CTC A). Simplex PCR was performed for DNA amplification at a final volume of 25 μl which consisted of 12.5 μl of 2× PCR master mix (Green Master, Promega, USA), 150 ng of the DNA template, 1 μl of each primer (10 pmole), and up to 25 μl nuclease-free water were mixed in a PCR tube.

The amplification was performed in a programmable heating block, (Gradient Thermal Cycler, Veriti Applied Biosystem, USA) at 95°C for 10 min, followed by 35 cycles were run under the following conditions; denaturation at 95°C for 30 s, annealing at 50°C for 30 s, and extension at 72°C for 30 s. After the final cycle, the preparations were kept for 10 min at 72°C as a final extension [18].

### Gel electrophoresis

PCR products were electrophoresed in 1% agarose gel (GX 040.90, Gen AGarose, L.E., Standard DNA/RNA agarose, Molecular Biology Grade, Inno-Train Diagnostik, D–61476, Kronberg/Taunus) containing ethidium bromide as 1 µl/ml electrophoresis buffer at 100 V for 60 min. Using 100 bp DNA ladder in (SCiE–PLAS, HU 10, 5636, UK). Then, the results were obtained through high-performance ultraviolet transilluminator, (UV, INC, UK). The image of the PCR products containing the positive DNA sequence of 360 bp was amplified using (Biodoc Analyzer software, Biometra, Germany).

### Restriction enzyme digestion

The PCR product of *cyt b* gene was subject to restriction enzyme Hinf I. 1 μl from the enzyme (Biolab, Canada) with 1 μl from the 1× reaction buffer were applied to 8 μl of PCR product. The digestion mixture was incubated for 3 h at 37°C (According to enzyme manual). The digested products were separated by electrophoresis in 1% agarose gel in TBE buffer and visualized by UV transillumination and analyzed using Gel Documentation System (DOC–It^®^ LS, Image acquisition software).

### Statistical analysis

The prevalence of adulteration of buffalo’s milk samples by adding cow milk was calculated by dividing the number of adulterated samples by the total number of the examined samples. Data were entered into Microsoft Excel Spreadsheet.

## Results

Unfortunately, the majority of the examined raw buffalo’s milk collected from Assiut City markets were mixed with bovine milk. From the examined 50 raw buffalo’s milk samples, a very high percentage (90%) was found to be adulterated with cow’s milk, while, only 10% of the samples free from adulteration (Table-1).

**Table-1:** Prevalence of adulteration of buffalo’s milk samples by adding cow milk.

Number of examined samples	n (%)	

	Pure buffalo samples	Adulterated samples
50	5 (10)	45 (90)

**Figure-1:**
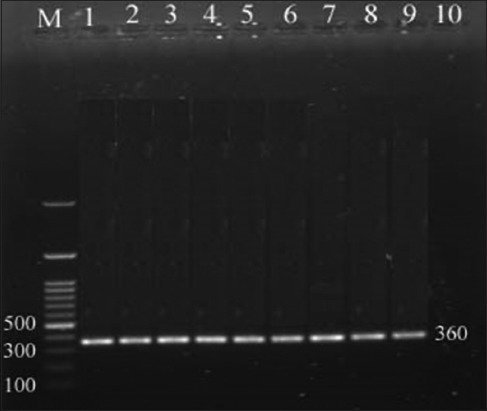
Electrophoretic analysis of polymerase chain reaction products amplified with *cytochrome b (cyt b)* gene. Lane (M) DNA ladder100 bp; lanes (1-8) positive random milk samples with specific bands at 360 bp; lane (9) positive control of *cyt b* gene; and lane (10) negative control using *E. coli* bacteria.

**Figure-2:**
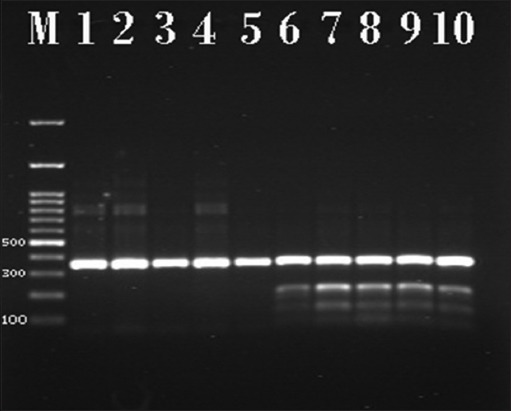
Polymerase chain reaction products of *cytochrome b (cyt b)* gene after treated by Hinf I enzyme of selected milk samples visualized on agarose gel electrophoresis. Lane (M) DNA ladder 100 bp; lanes (1-5) nature buffalo’s milk samples with specific bands at 360 bp; and lane (6-10) adulterated milk samples by cows’ milk with specific bands at 360, 210, and 150 bp.

In this study, the primers used had detected the *cyt b* gene, and the amplicons size was 360 bp as shown in Figure-1. Hinf I enzyme can cleaved the amplicon; the fragment size differs according to species. In case of bovine milk, the digested PCR product was divided into the following 360, 210, and 150 bp. On the other side, in the buffalo’s milk, the Hinf I enzyme cannot digest the amplicon product and the fragment still at 360 bp (Table-2 and Figure-2).

**Table-2:** Fragment length for cow and buffalo species after digestion of PCR products (360 bp) with Hinf I enzyme.

Animal species	Fragment length bp
Buffalo	360
Cow	360, 210, and 150

PCR=Polymerase chain reaction

## Discussion

Identifying milk species sold in the markets and used in the manufacture of milk products is a critical point in the quality control measures.

It is apparent from the analysis of the tested samples that a large number of samples procured did not conform to the legal standards prescribed by the Food Safety and Standards Authority of Egypt. Moreover, these results show deceitful practice which can affect the consumer rights, due to economics and the risk of consumption of unknown origin of milk [19]. Despite food legislation, adulteration remains uncontrolled and legal steps laid down in the act are extremely difficult to maintain due to inadequate and untrained workforce and laboratory facilities.

The results obtained in this study were higher than that postulated by Abdelfatah *et al*. [18] and Zarei *et al*. [20]; they indicated that the percentage of adulterated samples was 50% and 70%, respectively. The high incidence of adulteration in this study in comparison with the previous studies due to the difference between the locations of the studies, in addition, the low numbers of buffalos and the low buffalo’s milk production in Assiut in compare with Mansoura, Egypt, and Iran.

There are many methods have been used for identification of species origin of raw milk as chemical, immunological, and molecular techniques. There are several molecular techniques namely, PCR-RFLP [18], multiplex PCR [14] and Real-time PCR [21], and DNA based fluorometric method [22]. These PCR techniques can be used to differentiate between the closed related species.

The *cyt b* gene was reported to be highly polymorphic and could be used to differentiate between the buffalo and cow species [23]. In RFLP technique, the amplified PCR is broken to different size fragment according to the restriction enzyme used in the assay. On a previous study [18] evaluated four different restriction enzymes (Hinf I, Hind III, Hae III, and Bsa I) were evaluated to differentiate between the buffalo’s and cow’s milk and they concluded that the Hinf I enzyme was cleaved the amplicons of *cyt b*, but the other enzymes could not digest the amplified gene. In addition, the authors tried to estimate the sensitivity of RFLP-PCR method for detecting cow’s milk in buffalo’s one by made a mixture of different cow milk percentages 50, 40, 30, 20, 10, 5, 1, and 0.5% and they found that the lowest percentage of cow’s milk could be detected by RFLP assay was 5%.

However, there are many studies carried out the detection limit of PCR in addition of cow’s milk to buffalo’s one such as 0.5% [24,25] or minimum level at 0.1% [26].

## Conclusion

The results obtained in this study declared the high percentage of adulteration of buffalo’s milk by cow’s one. Thus, reflecting the fraudulent practice in raw buffalo’s milk sold in Assiut markets, it is recommended to the authorities to monitor the milk sold in the markets to prevent the buffalo’s milk adulteration by cow’s milk. Moreover, we recommended to increase the studies about milk adulteration using different molecular techniques to detect the most accurate and sensitive technique to use as routine testing to detect and avoid such type of milk adulteration. In addition, PCR-RFLP method used in this study was a useful and straightforward approach for detection buffalo’s milk adulteration.

## Author’s Contributions

RME and DSMAE designed the plan of work experiment. RME carried out the laboratory work and analyzed the results. Both authors drafted, read and approved the final manuscript.
